# Linking Inflammation to Natural Killer T Cell Activation

**DOI:** 10.1371/journal.pbio.1000226

**Published:** 2009-10-27

**Authors:** Mariolina Salio, Vincenzo Cerundolo

**Affiliations:** Nuffield Department of Clinical Medicine, Weatherall Institute of Molecular Medicine, University of Oxford, Oxford, United Kingdom

## Abstract

Immune activation is often associated with inflammation, but inflammation's role in the expansion of antigen-specific immune responses remains unclear. This primer focuses on recent findings that show how specific natural killer T cells are activated by inflammatory messengers, thus illuminating the cellular and molecular links between immunity and inflammation.

To help us fight infections, the immune system relies on the coordinated activity of innate and adaptive immunity. Innate immunity represents an evolutionarily conserved first line of defense, kicking into action within hours of encountering pathogens, while adaptive immunity involves a higher degree of specificity for pathogen-encoded molecular determinants and long-term memory responses. Components of the innate immune system include epithelial barriers, antimicrobial compounds, and a range of different cell types, including dendritic cells, macrophages, neutrophils, and natural killer cells. Conversely, the main components of adaptive immunity are B and T lymphocytes, which survey billions of antigens via cell surface receptors that undergo somatic DNA rearrangements to confer almost unlimited specificities. Activation of innate responses, triggered by the detection of conserved microbial molecules (such as viral nucleic acids or bacterial cell wall components) by pattern-recognition receptors (such as Toll-like receptors), is critical for eliciting antigen-specific immune responses [1]. Over the past ten years it has emerged that a population of unconventional T lymphocytes, called natural killer T cells (NKT cells), operates at the interface between innate and adaptive immune responses; these cells express rearranged antigen-specific surface T cell receptors (although with limited diversity), but are also capable of a very rapid response mode, resulting in the activation of antigen presenting cells and facilitating the development of adaptive immunity.

Whereas most T lymphocytes recognize peptide antigens presented by molecules of the major histocompatibility complex (MHC), NKT cells recognize lipid antigens presented by CD1d molecules, which are functionally related to MHC molecules [2]. Upon activation, NKT cells modulate the activity of CD1d-expressing cells via the costimulatory molecule CD40 ligand and induce interferon (IFN)-γ-dependent activation of other cell types, ultimately enhancing antigen specific B and T cell responses (Figure 1) [3]. Because of their effect on multiple cells of the immune system, NKT cells contribute to the regulation of a variety of processes, such as self-tolerance, tumor surveillance, and anti-microbial responses [4].

**Figure 1:**
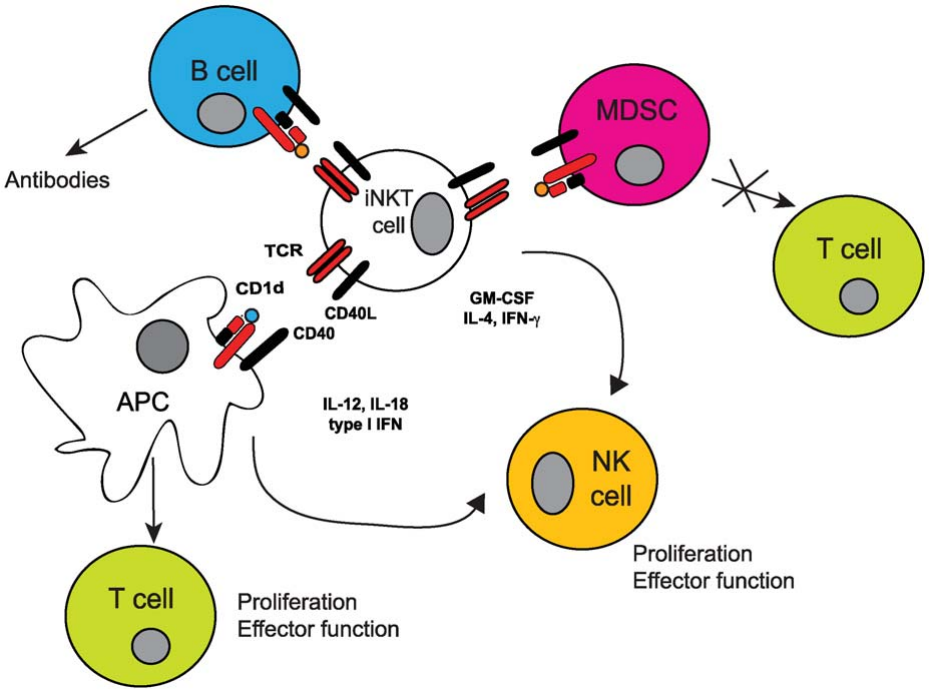
iNKT cells at the interface between innate and adaptive immunity. iNKT cells recognize lipids presented by CD1d molecules. Upon activation, iNKT cells modulate the function of CD1d-expressing cells, such as APCs and B cells. This leads to the priming of antigen-specific T cells, induction of antibody responses, and activation of natural killer cells, a subset of cells acting in the innate immune response. iNKT cells can also inhibit the suppressive function of MDSCs.

## Auto-Reactivity by Design

During maturation of T lymphocytes the majority of auto-reactive cells are destroyed in the thymus to prevent autoimmunity. However, a proportion of NKT cells expressing a semi-invariant T cell receptor, hereafter referred to as invariant NKT cells (iNKT cells), maintain the ability to recognize a range of endogenous lipids in the context of CD1d molecules during both inflammatory and non-inflammatory conditions. It has recently been shown that in the absence of inflammation, iNKT-cell auto-reactivity leads to a special activation state, characterized by impaired calcium signaling, which leads to the secretion of GM-CSF (granulocyte-macrophage colony-stimulating factor), with limited amounts of inflammatory cytokines [5]. In contrast, during microbial infection, inflammatory cytokines, such as interleukin (IL)-12 and IL-18, enhance basal iNKT-cell auto-reactivity and promote secretion of IFN-γ [6]–[9]. In addition, during inflammatory responses, iNKT-cell activation can be influenced by increased expression of surface CD1d molecules by activated antigen presenting cells (APCs) [10],[11] and/or by increased expression of enzymes leading to the biosynthesis of endogenous self lipids [7],[8]. These multiple mechanisms influencing iNKT-cell activation suggest an important link between inflammation and iNKT cells. Indeed, recent results have highlighted the ability of iNKT cells to abolish the suppressive activity of myeloid-derived suppressor cells (MDSCs), which are expanded during tumor growth and microbial infections and restore antigen-specific immune responses during influenza virus infection (Figure 1) [12].

Although the identity of several bacteria-derived lipids capable of activating iNKT cells has been determined [6],[13],[14], the identity of the lipids modulating iNKT-cell auto-reactivity has been elusive and is currently being investigated by several groups [15]. Identification of such lipids is important to a fuller understanding of the rules regulating CD1d assembly and loading, and also for the design of novel compounds that can selectively modulate iNKT-cell activation [16],[17]. Results published in this issue of *PLoS Biology* [18], and in earlier issues of *PLoS ONE* [19] and *The Journal of Immunology* [20], shed new light on the identity of iNKT cell natural ligands and provide further support for the link between inflammation and iNKT-cell activation.

## A Closer Look at the Lipid Repertoire Bound to CD1d Molecules

Analysis of the ability of defined lipids to stimulate iNKT-cell activation has revealed that a restricted number of murine iNKT-cell clones can recognize phosphatidylinositol (PI) [21], phosphatidylethanolamine (PE), and phosphatidylglycerol (PG) and the ganglioside GM3 [22]. However, reactivity to these lipids is weak and limited to a few iNKT-cell clones. To identify other endogenous natural iNKT-cell agonists, one approach has relied on the use of CD1d^+^ cells with defined genetic defects in the lipid biosynthetic pathway. The results of these experiments have shown that a class of lipids known as glycosphingolipids (GSL), such as isoglobotrihexosylceramide (iGb3), contributes to the pool of endogenous lipids that leads to human and murine iNKT cell activation [6],[23],[24]. However, the presence of iGb3 in dendritic cells and developing T lymphocytes remains controversial [25]–[29]. Furthermore, it has become clear that experiments carried out in mice or in cells with defective GSL catabolism need to be carefully controlled, because impaired iNKT-cell positive selection and activation could be due not to the absence of activating lipids, but to the failure of lipid presentation resulting from sequestering of lipids in the lysosomes of these APCs [30],[31]. Consistent with this possibility, mice deficient in the lysosomal enzyme β-hexosaminidase, which generates iGb3 from iGb4, have impaired iNKT-cell development [24], whereas iGb3 synthase-deficient mice have normal numbers of iNKT cells [25]. Thus, the role of iGb3 as a self antigen for iNKT cells remains unclear.

In order to better characterize the classes of self-lipids available for recognition by iNKT cells, two groups have independently employed a novel approach to analyze the repertoire of lipids bound to CD1d molecules [18]–[20]. The Cresswell group engineered human CD1d molecules that could be proteolytically cleaved after recycling physiologically through the endocytic pathway and then compared lipids that were associated with CD1d molecules in different cellular compartments, that is, either retained in the endoplasmic reticulum (ER), secreted in soluble form, or cleaved [20]. By using multiple mass spectrometry methods to identify the major lipid species, the researchers reported that the ER-retained form of CD1d was predominantly loaded with phosphatidylcholine (PC), the most abundant phospholipid in eukaryotic cells. The only detectable lipid associated with the secreted CD1d molecules was sphingomyelin, which is synthesized in the Golgi and is not present in the ER, whereas the protease-cleaved CD1d molecules were loaded with PC, sphingomyelin, and lysosphospholipids. In contrast, Gumperz and colleagues focused their analysis on lipids associated with secreted human CD1d molecules, as they had previously observed that, in contrast to mouse iNKT cells, autoreactivity of human iNKT-cell clones is largely independent of CD1d endosomal trafficking [32]. In the *PLoS ONE* paper, Gumperz and colleagues found a large spectrum of lipids associated with soluble human CD1d molecules [19]. By a high-resolution analysis, a total of 177 lipid species were identified, comprising glycerophospholipids (including common diacylglycerol species, plasmalogens, lysophospholipids, and cardiolipins) and sphingolipids (including sphingomyelins and GSL, such as the ganglioside GM3). Altogether, these results highlight the possibility that, depending on the cellular localization, CD1d molecules may be loaded with a different spectrum of endogenous ligands.

**Figure 2:**
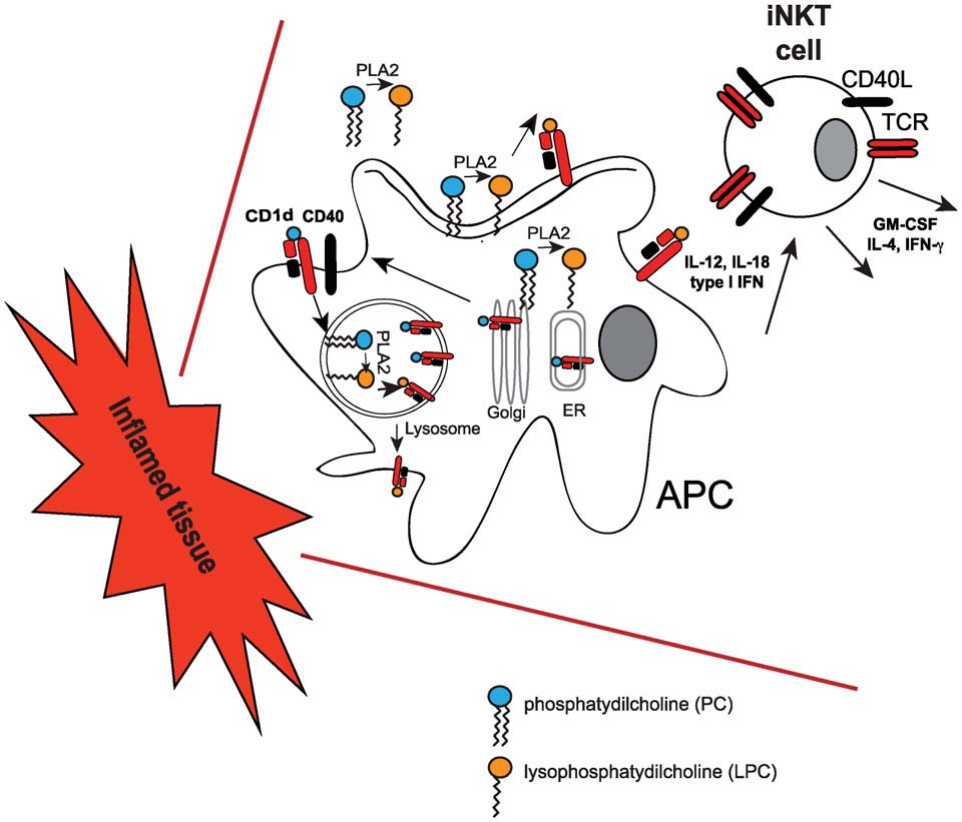
iNKT-cell activation by lysosphospholipids. During inflammation cytoplasmic, membrane and secreted phospholipases (such as PLA2) produce lysophospholipids (such as LPC) from cellular phospholipids. Lysosphospholipids can be loaded onto CD1d molecules at the cell surface, in the lysosomes, or during intracellular trafficking through the ER and the Golgi. CD1d-LPC complexes elicit iNKT-cell activation in concert with IL-12, IL-18, and type I IFN secreted by APCs during inflammatory reactions.

Given that some of these lipids are known to be bioactive and have been reported to play significant roles in cancer, autoimmune disease, cellular signaling, and cell death [33],[34], the Gumperz group has investigated the ability of iNKT cells to recognize and react to synthetic preparations of all the CD1d bound lipids - these findings are reported in this issue of *PLoS Biology* [18]. Remarkably, the mono acyl lysophosphatidylcholine (LPC) (Figure 2) was the only antigenic species capable of activating both a panel of iNKT-cell clones and lines and, albeit weakly, freshly isolated peripheral blood lymphocytes.

## LPC as an Inflammatory Lipid and Stimulating Signal for iNKT Cells

LPC is produced by the phospholipase A2 enzymes (PLA2), which can be localized to a number of intracellular and extracellular sites. Activation of PLA2 by a variety of growth factors, hormones, and cytokines can lead to the release of LPC into the cytoplasm, the lysosome, or at the cell surface [35]. At all these locations, LPC could be available for loading onto CD1d molecules (Figure 2). Of relevance is the observation from the Gumperz group that blocking secreted PLA2 activity in monocytes (and thereby reducing the levels of lipid ligand) with a polyclonal antibody, led to reduced basal iNKT-cell activation without affecting CD1d expression [18]. Since LPC accumulates to high concentrations in blood and other fluids during chronic inflammation, and since Gumperz and colleagues showed that lysophospholipids can bind to CD1d molecules previously loaded with other cellular ligands or GSL [18], monitoring the levels of CD1d-bound LPC could represent one of the mechanisms leading to iNKT-cell activation and expansion. Interestingly, Dhodapkar and colleagues previously reported an increase of LPC species in one pathological setting - in the plasma of myeloma patients. This was accompanied by an expansion of a subset of NKT cells [36], which, unlike iNKT cells, express a broader range of T cell receptors. These results suggest that recruitment and expansion of invariant and non-invariant NKT cells could occur more widely in different inflammatory settings and eventually contribute to immune pathology. Indeed, iNKT cells have a chemokine receptor profile that allows them to preferentially “home” to inflamed tissues [37]. It is tempting to speculate that, during inflammation, secreted LPC could be presented by APCs recruited at inflammatory sites, resulting in iNKT-cell activation (Figure 2). However, the efficiency of LPC presentation in vivo remains to be defined. Gumperz and colleagues provide some support for this notion by showing very weak iNKT-cell stimulation with APCs expressing wild-type CD1d molecules. In contrast, the authors show that recognition of LPC by iNKT cells was enhanced using CD1d molecules unable to recycle from the cell surface to the lysosomes (where the acidic environment could lead to dissociation of the CD1d-lipid complexes) [18]. In addition, higher concentrations of LPC failed to activate iNKT-cell clones, showing an unexplained inhibitory effect, which could be due to the formation of micelles or less–CD1d-accessible structures [18].

It is known that the length of the lipid hydrocarbon chains determines the stability of lipid binding to CD1d molecules, which in turn influences iNKT-cell activation [38]. Although further studies are warranted to determine the binding affinity of lysophospholipids for CD1d molecules and the half-life of these complexes, it is likely that the mono alkyl chain LPC will have a higher rate of dissociation from CD1d molecules than the dual alkyl chain PC. Thus, the combination of limited presentation by recycling CD1d molecules, with the inhibitory effect of high LPC concentrations and possibly the short half-life of CD1d-LPC complexes, could be important features that help to fine-tune iNKT-cell responses in the context of prolonged inflammatory processes.

The crystal structures of human and murine CD1d molecules have revealed the presence of two hydrophobic channels, A′ and C′, which are occupied by the lipid tails of iNKT-cell agonists [39]. Interestingly, the results by the Gumperz laboratory in this issue of *PLoS Biology* have highlighted a dichotomy in the ability of LPC and PC to stimulate iNKT cells [18]. It will be, therefore, very informative to carry out structural studies comparing CD1d molecules loaded with either lysophospholipids or phospholipids (e.g., mono versus di acyl lipids) to assess whether the differential ability of LPC and PC to activate iNKT cells may be accounted for by variations in the orientation of the polar head as a consequence of different binding of their lipids chains to the A′ and C′ hydrophobic channels. This possibility would be consistent with previously published CD1d structures, revealing the ability of different phospholipids to bind CD1d molecules in different orientations [40],[41].

In conclusion, the identification of LPC as an endogenous ligand for iNKT cells is an important finding for the understanding of the role that iNKT cells and other immune cells play during inflammation. It will also be interesting to correlate iNKT-cell numbers and activation with changes in activity of the PLA2 isoforms during different inflammatory conditions (for example, upon microbial infections and Toll-like receptor-mediated activation of APCs or during chronic inflammatory processes, such as cancer). Future studies will reveal whether analogues of lysophospholipids could be exploited as novel adjuvants to further harness iNKT cells' ability to bridge innate and adaptive immune responses or to fine-tune iNKT-cell autoreactivity during autoimmune diseases.

## Abbreviations

APCs: antigen presenting cells
ER: endoplasmic reticulum
GM-CSF: granulocyte-macrophage colony-stimulating factor
GSL: glycosphingolipid
IFN: interferon
IL: interleukin
iNKT cells: invariant NKT cells
LPC: lysophosphatidylcholine
MDSCs: myeloid-derived suppressor cells
MHC: Major Histocompatibility Complex
NKT cells: natural killer T cells
PC: phosphatidylcholine
PE: phosphatidylethanolamine
PG: phosphatidylglycerol
PI: phosphatidylinositol
PLA2: phospholipase A2
